# Characteristics of Pediatric Antimicrobial Stewardship Programs: Current Status of the Sharing Antimicrobial Reports for Pediatric Stewardship (SHARPS) Collaborative

**DOI:** 10.3390/antibiotics7010004

**Published:** 2018-01-25

**Authors:** Christopher McPherson, Brian R. Lee, Cindy Terrill, Adam L. Hersh, Jeffrey S. Gerber, Matthew P. Kronman, Jason G. Newland

**Affiliations:** 1 Department of Pharmacy, St. Louis Children’s Hospital, 1 Children’s Place, St. Louis, MO 63110, USA; mcpherson_c@kids.wustl.edu; 2 Department of Pediatrics, Washington University School of Medicine, 660 S Euclid Ave, St. Louis, MO 63110, USA; 3 Division of Pediatric Infectious Diseases and Health Services and Outcomes Research, Children’s Mercy Hospital and Clinics, 2401 Gillham Rd., Kansas City, MO 64108, USA; blee@cmh.edu; 4 Division of Pediatric Infectious Diseases, Washington University School of Medicine, 660 S Euclid Ave, St. Louis, MO 63110, USA; terrill@wustl.edu; 5 Division of Pediatric Infectious Diseases, Department of Pediatrics, University of Utah School of Medicine, 295 Chipeta Way, Salt Lake City, UT 84108, USA; adam.hersh@hsc.utah.edu; 6 Division of Infectious Diseases, Children’s Hospital of Philadelphia, 3401 Civic Center Blvd, Philadelphia, PA 19104, USA; gerberj@email.chop.edu; 7 Department of Biostatistics and Epidemiology, Perelman School of Medicine of the University of Pennsylvania, 3400 Civic Center Blvd, Philadelphia, PA 19104, USA; 8 Division of Pediatric Infectious Diseases, Department of Pediatrics, University of Washington School of Medicine, Seattle Children’s Hospital 4800 Sand Point Way NE, M/S MA.7.226, Seattle, WA 98105, USA; matthew.kronman@seattlechildrens.org

**Keywords:** antibiotic, antimicrobial stewardship, quality improvement

## Abstract

In response to the growing epidemic of antibiotic-resistant bacterial infections, antimicrobial stewardship programs (ASP) have been rapidly implemented in the United States (US). This study examines the prevalence of the Centers for Disease Control and Prevention’s (CDC) seven core elements of a successful ASP within a large subset of US Children’s Hospitals. In 2016, a survey was conducted of 52 pediatric hospitals assessing the presence of the seven core elements: leadership commitment, accountability, drug expertise, action, tracking, reporting, and education. Forty-nine hospitals (94%) had established ASPs and 41 hospitals (79%) included all seven core elements. Physician accountability (87%) and a dedicated ASP pharmacist or drug expert (88%) were present in the vast majority of hospitals. However, substantial variability existed in the financial support allotted to these positions. This variability did not predict program actions, tracking, reporting, and education. When compared with previous surveys, these results document a dramatic increase in the prevalence and resources of pediatric stewardship programs, although continued expansion is warranted. Further research is required to understand the feasibility of various core stewardship activities and the impact on patient outcomes in the setting of finite resources.

## 1. Introduction

Increasing rates of drug-resistant bacterial infections have led to an international effort to ensure appropriate antimicrobial utilization [1]. In the United States, the Infectious Diseases Society of America, supported by the Pediatric Infectious Diseases Society and American Academy of Pediatrics, have identified antimicrobial stewardship programs (ASPs) as essential components of this effort [2]. In fact, the Joint Commission now requires all acute care hospitals to have ASPs for accreditation [3]. In response, pediatric ASPs have increased in prevalence [4,5]. 

To ensure optimal stewardship efforts, the Centers for Disease Control and Prevention (CDC) has identified seven core elements of a successful program [6]. In the setting of rapid implementation of pediatric ASPs across the nation, it is essential to ensure best practices and establish benchmarks. The Sharing Antimicrobial Reports for Pediatric Stewardship (SHARPS) collaborative was established for this purpose [7]. The collaborative enrolls interested hospitals with a pharmacist and/or physician representative dedicated to antimicrobial stewardship; however, hospitals vary with regard to stewardship resources and efforts. Initially comprised of a large subset of free-standing pediatric hospitals in the United States, the collaborative has expanded to include designated pediatric hospitals within general hospitals. Recently, each site completed a survey to describe the resources and efforts of their ASP. The purpose of this study is to describe the current status of a large cohort of pediatric ASPs with respect to the seven core elements identified by the CDC.

## 2. Results

Surveys were completed describing 52 hospitals by 26 pediatric infectious diseases physicians (50%) and 26 pharmacists (50%) between June 2016 and July 2017. Participant hospitals had a median of 269 licensed beds (range 80–628) with 11,417 admissions per year (range 2000–37,556). At the time of survey, 49 hospitals (94%) had established a formal ASP. Forty-one hospitals (79%) included all seven core elements described by the CDC. The element with lowest compliance was accountability (the presence of a physician leader responsible for program outcomes) at 87% (Table 1).

In programs with dedicated leadership from a physician(s), the median supported effort was 0.3 full time equivalents (FTEs) (Table 2).

Drug expertise from a dedicated pharmacist(s) was slightly more prevalent than dedicated leadership from a physician(s) (Table 1), with a median of 1 FTEs (Table 2). Among the 52 hospitals, prospective audit with feedback and prior approval was implemented in 29 (56%), prospective-audit with feedback alone in 10 (19%) and prior approval alone in 9 (17%). Forty-six hospitals (88%) utilized clinical practice guidelines in their programs. The vast majority of hospitals (>90%) were tracking antibiotic prescribing and/or resistance patterns and regularly reporting outcomes and providing education to staff. 

Despite similar bed counts and admission volumes, hospitals meeting all seven core elements described by the CDC committed significantly more FTEs to physicians and pharmacists (Table 3). There was no difference in the number of drugs prospectively audited or requiring prior authorization between programs with and without all seven core elements. There was also no difference in the proportion monitoring specific outcomes of antimicrobial stewardship. 

## 3. Discussion

This study documents the high prevalence and robust nature of ASPs in hospitals caring for pediatric patients. The majority of programs represented by the SHARPS collaborative comprise all seven core elements of a successful ASP described by the CDC. However, significant variability in resources exists among ASPs. The ongoing expansion and success of ASPs will rely on critical evaluation of resources needed to conduct the activities with the highest clinical and economic impact.

In the context of previous surveys, this study provides evidence that rapid growth has occurred in both the number and size of inpatient ASPs serving pediatric patients. In mid-2011, sixteen of 38 (42%) free-standing children’s hospitals surveyed had established a formal ASP [5]. By 2015, thirty of the 36 members of the SHARPS collaborative (83%) had formalized antimicrobial stewardship efforts [7]. The surveys reported in this study, completed between summer 2016 and summer 2017, document an increase in collaborative membership (*n* = 52), with a higher proportion of those centers having established ASPs with dedicated financial resources (94%). 

The human resources devoted to antimicrobial stewardship among these hospitals has continued to increase. In 2011, programs had a median of 0.63 total FTEs [5]. The larger 2015 survey of the SHARPS collaborative documented no change in the median FTEs devoted to ASPs [7]. The present survey notes a doubling of overall support, to a median of 1.4 total FTEs. Specifically, a pharmacist(s) devoted to ASP is (are) now present at 88% of surveyed hospitals, compared to 34% in 2011 [5]. Pharmacist FTEs increased from a median of 0.5 in 2011 to a median of 1 in the current survey. In addition, partial salary support for an ASP physician was 87% for the current survey versus only 37% of pediatric hospitals in 2011. We did not note an increase in median FTEs devoted to physicians between 2011 and the present, although one may be warranted given the vital synergy between accountable physicians, drug experts and other members of the stewardship team. Similarly, while 6% of pediatric ASPs supported a data analyst in mid-2011, the current study documents a substantial increase to 35% of programs. This increase highlights progress in the valuation of process and outcome data to successful antimicrobial stewardship, although this expertise remains deficient in the majority of programs. 

This study found no significant difference between hospitals with and without all seven core CDC elements with regard to the quantified stewardship activities. The intrinsic difference in FTEs between these classifications raises additional questions. Contrary to a previous survey, this study does not support a correlation between FTEs and the number of antibiotics monitored or restricted [5]. Similarly, programs with fewer human resources do not appear to have less robust monitoring of outcomes. Although this could represent a saturation point at which additional FTEs devoted toward stewardship do not generate additional activity, it more likely represents the novelty of many programs/positions captured in our survey and a deficiency in our measures of ASP activity. Additionally, our survey only captured a snapshot of broad stewardship efforts. Previous studies have documented the positive impacts of ASPs [4]; future research must attempt to quantify the direct impact associated with specific stewardship interventions on antibiotic use and patient outcomes. These studies will assist ASPs in directing limited resources to the most effective strategies. Fortunately, the SHARPS collaborative is uniquely positioned to undertake these studies through recruitment of a large, geographically diverse cohort of pediatric ASPs.

This study has important limitations. Although the survey evaluated for the presence of several common antimicrobial stewardship interventions and outcome measures, the survey did not comprehensively evaluate all aspects of each ASP nor the method and intensity of each activity or core element. For example, we did not evaluate the programs based on the numbers of antimicrobials prospectively audited and/or restricted by an ASP. Additionally, we did not distinguish the level of compliance with each core element. For example, publishing an antibiogram was the most common element of tracking (92%); however, a smaller proportion of ASPs support a data analyst (35%) and/or track antimicrobial prescribing (79%). Finally, SHARPS is a voluntary collaborative comprised mainly of academic, free-standing pediatric hospitals in urban settings. Participation requires at least one dedicated member of an ASP. Therefore, our results likely overestimate the resources and efforts of pediatric ASPs nationally.

## 4. Materials and Methods 

The SHARPS collaborative was established in the fall of 2013 and currently includes 52 hospitals caring for pediatric patients. From June 2016 to July 2017, the pharmacist and/or physician representative of current and new member hospitals were surveyed electronically with regard to hospital demographic data, FTEs devoted to antimicrobial stewardship, and stewardship activities (Supplementary Materials File 1). Survey design and development have been previously described [5]. 

A formal ASP was defined by the presence of institutional support for a dedicated physician, pharmacist, and/or data analyst who monitors antimicrobials. Compliance with the CDC’s seven core elements of hospital ASPs was determined by responses to specific survey elements. Leadership commitment, accountability and drug expertise were determined by the presence of FTEs dedicated to any antimicrobial stewardship personnel, a physician, and pharmacist, respectively. Action was determined by the presence of prospective-audit with feedback, formulary restriction of antimicrobials, clinical practice guidelines, an intravenous to oral switch program, and/or automatic stop orders for empiric antimicrobials. The presence of data analyst FTEs dedicated to antimicrobial stewardship and/or regular review of antimicrobial use and/or drug resistance confirmed tracking was performed. Reporting was present if a hospital regularly shared antimicrobial use data with hospital staff and/or distributed an annual antibiogram. Education was determined by the presence of educational activities for prescribers, trainees, and/or patients and families.

Statistical analyses were performed using IBM SPSS Statistics, version 22. Descriptive statistics were utilized to quantify hospital demographics and FTEs dedicated to antimicrobial stewardship. The Mann-Whitney U test or Fisher’s exact test were utilized to compare hospital demographics, FTEs, stewardship activities, and outcomes monitored at hospitals with and without the presence of all seven core elements. *p* values less than 0.05 were considered statistically significant.

## 5. Conclusions

A large number of pediatric hospitals have implemented ASPs, with the majority comprised of all seven core elements recommended by the CDC. The most common missing elements were dedicated leadership from a physician and drug expertise from a pharmacist, highlighting the importance of ongoing efforts quantifying successful antimicrobial stewardship activities and patient-level impact. These efforts will guide the allocation of limited resources, and may also justify the expansion of these vital programs.

## Figures and Tables

**Table 1:** Compliance with seven core elements of a hospital antimicrobial stewardship program (ASP).

Core Element	Compliance ^1^ (*n* = 52)
Leadership Commitment	49 (94%)
Accountability	45 (87%)
Drug Expertise	46 (88%)
Action	51 (98%)
Tracking	51 (98%)
Reporting	51 (98%)
Education	48 (92%)

^1^ Compliance reported as *n* (%).

**Table 2:** Full time equivalents (FTEs) devoted to antimicrobial stewardship by position.

Position	Full Time Equivalents ^1^
Physician, *n* = 45	0.3 (0.2–0.5)
Pharmacist, *n* = 46	1 (0.5–1)
Data analyst, *n* = 18	0.5 (0.2–0.5)
Infection preventionist, *n* = 7	1 (0.5–2)
Total	1.4 (0.8–2)

^1^ Full time equivalents reported as median (interquartile range).

**Table 3:** Demographic Data of Hospitals stratified by Compliance with Centers for Disease Control and Prevention’s (CDC) Core Elements.

Variable	Hospitals with Full Compliance (*n* = 41)	Hospitals with One or More Missing Elements (*n* = 11)	*p* Value
**Demographics**			
Hospital beds ^1^	270 (200–353)	256 (189–337)	0.94
Admissions per year ^1^	11,417 (7417–15,458)	10,065 (5013–29,276)	1
Teaching hospital ^2^	38 (93%)	11 (100%)	1
Freestanding children’s hospital ^2^	27 (66%)	7 (64%)	1
**Human resources ^1^**			
Physician FTEs ^3^	0.3 (0.3–0.5)	0 (0–0.2)	0.001
Pharmacist FTEs	1 (0.5–1)	0 (0–1)	0.007
Data analyst FTEs	0 (0–0.3)	0	0.21
Infection preventionist FTEs	0	0 (0–0.5)	0.14
**Stewardship activities ^1^**			
Drugs prospectively audited	3 (0–14)	5 (0–11)	0.86
Drugs restricted	7 (0–15)	6 (0–12)	0.90
**Outcomes Monitored ^2^**			
Antimicrobial use	32 (78%)	9 (82%)	1
Antimicrobial costs	23 (56%)	8 (73%)	0.49
Antimicrobial resistance	33 (80%)	9 (82%)	1
Rate of *C. difficile* infection	36 (88%)	10 (91%)	1
Rate of antimicrobial ADRs ^4^	23 (56%)	7 (64%)	0.74

^1^ Values reported as median (interquartile range). ^2^ Values reported as *n* (%). ^3^ FTEs = full-time equivalents. ^4^ ADRs = adverse drug reactions.

## Supplementary Materials

The following are available online at http://www.mdpi.com/2079-6382/7/1/4/s1, File 1: SHARP Intake Questionnaire.
