# Supplementary material for: Characteristics of Pediatric Antimicrobial Stewardship Programs: Current Status of the Sharing Antimicrobial Reports for Pediatric Stewardship (SHARPS) Collaborative
Source: Antibiotics (Basel). 2018 Jan 25;7(1):4. doi: 10.3390/antibiotics7010004 (PMC5872115; doi:10.3390/antibiotics7010004)
Supplement: Supplementary file 1 [file antibiotics-07-00004-s001.pdf]

SHARP Intake Questionnaire

Please complete the survey below.

Thank you!

Hospital	◯ (AL) Children's of Alabama<br>◯ (MS) University of Mississippi Medical Center<br>◯ (AR) Arkansas Children's<br>◯ (CA) Rady Children's Hospital San Diego<br>◯ (CA) Lucile Packard Children's Hospital Stanford<br>◯ (CA) Children's Hospital of Los Angeles<br>◯ (CA) University of California San Francisco<br>◯ (CA) Valley Children's Healthcare<br>◯ (CO) Children's Hospital Colorado<br>◯ (CT) Connecticut Children's<br>◯ (DC) Children's National Medical Center<br>◯ (DE) Nemours-Alfred duPont<br>◯ (FL) Nicklaus Children's Hospital<br>◯ (FL) All Children's-St. Petersburg<br>◯ (FL) Nemours Children's Hospital - Orlando<br>◯ (FL) Holtz Children's Hospital<br>◯ (GA) Children's Healthcare of Atlanta<br>◯ (HI) Kapi'olani Medical Specialists<br>◯ (IL) Children's Hospital of Illinois<br>◯ (IL) Lurie Children's Hospital of Chicago<br>◯ (IL) St. Johns Children's Hospital - Springfield<br>◯ (IN) Riley Hospital for Children<br>◯ (KY) Kosair Children's Hospital<br>◯ (MA) Boston Children's<br>◯ (MI) Helen DeVos Children's<br>◯ (MI) University of Michigan<br>◯ (MN) Children's Hospital of Minnesota<br>◯ (MN) Mayo Clinic<br>◯ (MO) St. Louis Children's<br>◯ (MO) Children's Mercy Kansas City<br>◯ (MO) St. Louis University Hospital<br>◯ (MO) MU Women's and Children's Hospital<br>◯ (NC) Levine Children's Hospital<br>◯ (NE) Children's Hospital of Omaha<br>◯ (NJ) Bristol Myer Squib Children's<br>◯ (NJ) St. Barnabas Medical Center<br>◯ (NJ) Saint Peter's University Hospital<br>◯ (NY) Stony Brook Children's Hospital<br>◯ (NY) Buffalo Children's Hospital<br>◯ (NY) Cohen Children's Medical Center<br>◯ (OH) Akron Children's Hospital<br>◯ (OH) Nationwide Children's Hospital<br>◯ (OH) Rainbow Babies and Children's Hospital<br>◯ (OH) Cincinnati Children's<br>◯ (OR) Doernbecher Children's<br>◯ (PA) The Children's Hospital of Philadelphia<br>◯ (PA) Children's Hospital of Pittsburgh<br>◯ (SC) Medical University of South Carolina<br>◯ (TN) Le Bonheur Children's<br>◯ (TN) Monroe Carell Children's Hospital<br>◯ (TN) Vanderbilt University Medical Center<br>◯ (TN) St. Jude Children's Research Hospital<br>◯ (TX) Texas Children's<br>◯ (TX) Driscoll Children's Hospital - Corpus Christi<br>◯ (TX) Cook Children's - Fort Worth<br>◯ (TX) Dell Children's Medical Center<br>◯ (TX) Children's Hospital of San Antonio<br>◯ (TX) McLane Children's Scott & White Hospital<br>◯ (TX) University of Texas - Houston<br>◯ (UT) Primary Children's Hospital<br>◯ (WA) Seattle Children's Hospital<br>◯ (WI) American Family Children's - Madison<br>◯ (WI) Children's Hospital of Wisconsin

Responder Information

Name of the responder ________________________________

Email of the responder ________________________________

Role of the responder
- ◯ Pharmacist
- ◯ Physician
- ◯ Data analyst
- ◯ Other

Describe your role ________________________________

Hospital Information

Choose the option that best describes your hospital
- ◯ Freestanding children's hospital (with or without affiliated pediatric clinics)
- ◯ Children's hospital contained within a larger Adult/pediatric healthcare system
- ◯ Specialist Children's Hospital (e.g. Children's Cancer Center)
- ◯ Pediatric/Neonatal Units within an adult hospital
- ◯ Specialist Adult Hospital (e.g. cancer hospital) with a pediatric ward
- ◯ Other

Please describe your hospital ________________________________

Is your hospital a teaching hospital?
- ◯ Yes
- ◯ No

Please tell us the upper age limit for children admitted to your hospital. ________________________________

Total bed count (only pediatric beds if in a hospital that has adult beds) ________________________________

Total hospital admissions from 1/1/2015 to 12/31/2015 (only pediatric admissions if in a hospital with adult admissions) ________________________________

Pediatric Intensive Care

Does your center provide Pediatric Intensive Care (including invasive ventilation and inotropic support if required)?
- ◯ Yes
- ◯ No

Does your center provide Pediatric Surgical Intensive Care? (you do not have to have a separate unit)
- ◯ Yes
- ◯ No

Total PICU bed count ________________________________

Total Cardiac Intensive Care Unit (CICU) bed count ________________________________

How many PICU plus CICU (if applicable) admissions did your hospital have from 1/1/2015 to 12/31/2015? ________________________________

Neonatal Intensive Care

Does your center provide Neonatal Intensive Care?
◯ Yes
◯ No

Does your center provide Neonatal Surgical Intensive Care? (you do not have to have a separate unit)
◯ Yes
◯ No

Total NICU bed count ______________________________

How many NICU admissions did your hospital have from 1/1/2015 to 12/31/2015? ______________________________

Please provide some information about laboratory and imaging services at your hospital. (Please remember these questions are due to the global point prevalence aspect of this study)

Does your hospital have its own microbiology lab?
◯ Yes
◯ No

Is a majority of the virology testing performed in your microbiology lab?
◯ Yes
◯ No

Is a majority of the mycology testing performed in your microbiology lab?
◯ Yes
◯ No

Does your hospital have radiology services?
◯ Yes
◯ No

Do you have access in your hospital to CT scan?
◯ Yes, during daytime working hours
◯ Yes, all time
◯ No

Do you have access in your hospital to MRI scan?
◯ Yes, during daytime working hours
◯ Yes, all time
◯ No

Are Results From The Following Diagnostic Services Available Electronically?

	None	Some	All
Haematology	◯	◯	◯
Biochemistry	◯	◯	◯
Microbiology	◯	◯	◯
Radiology	◯	◯	◯

Assessment of the Antimicrobial Stewardship Program (ASP)- (These questions are to update our SHARPS data regarding all of our hospitals and to assess how our hospitals meet the Core Elements of ASP proposed by the CDC.)

Do you have a formal ASP? Y/N- Formal ASP is defined as monitoring antimicrobials and the institution provides financial support for a pharmacist, and/or physician, and/or data analyst?
- ◯ Yes
- ◯ No

Is your hospital in the process of developing a formal ASP?
- ◯ Yes
- ◯ No

Who does your ASP leaders report to?
- ☐ Quality and Safety
- ☐ Infectious Diseases Division
- ☐ Infection Control
- ☐ I Don't Know
- ☐ Other

(Check all that apply)

Describe the "other" ASP Leader.

____

Do you have an ASP policy that is approved by P&T and/or the medical executive committee?
- ◯ Yes
- ◯ No

Whom does your ASP serve?
- ◯ Children only
- ◯ Children and adults (e.g. joint ASP in a combined adult/pediatric hospital)

Does your hospital have a pharmacist(s) dedicated to ASP?
- ◯ Yes
- ◯ No

How many pharmacist FTEs are dedicated to the ASP?

____

Of the total pharmacy FTE, how much FTE is allocated to adult ID pharmacist?

____

Of the total pharmacy FTE, how much FTE is allocated to pediatric ID pharmacist?

____

Of the total pharmacy FTE, how much FTE is allocated to general adult pharmacist?

____

Of the total pharmacy FTE, how much FTE is allocated to general pediatric pharmacist?

____

Of the total pharmacy FTE, how much FTE is allocated to other types?

____

Describe the other pharmacy FTE type.

____

Does your hospital have service specific pharmacists who participate in your ASP program?
- ◯ Yes
- ◯ No

Describe how service specific pharmacists participate in your ASP program.

____

Does your hospital have a physician(s) dedicated to ASP?

◯ Yes
◯ No

How many physician FTEs are dedicated to the ASP? __________________________________

Of the total physician FTE, how much FTE is allocated to adult ID physician? __________________________________

Of the total physician FTE, how much FTE is allocated to pediatric ID physician? __________________________________

Of the total physician FTE, how much FTE is allocated to adult hospitalist? __________________________________

Of the total physician FTE, how much FTE is allocated to pediatric hospitalist? __________________________________

Of the total physician FTE, how much FTE is allocated to other? __________________________________

Describe the other physician FTE type.

____

Does your hospital have a data analyst dedicated to the ASP?

◯ Yes
◯ No

How many data analyst FTEs are dedicated to the ASP? __________________________________

Does your hospital have infection preventionists participate and help with your ASP?

◯ Yes
◯ No

How many infection preventionists FTEs are dedicated to the ASP? __________________________________

Are any of the following groups integrally involved with your ASP?

☐ Clinicians outside of ID
☐ Quality improvement staff
☐ Microbiology/Virology Staff
☐ Information Technology staff
☐ Nurses
(Check all that apply)

ASP Actions

Please respond to the following questions as they apply to pediatric patients only.

Do you perform prospective-audit with feedback?

◯ Yes
◯ No

Which of the following drugs do you perform audit with feedback?

- [ ] --------------------- All Antibiotics -------------------------
- [ ] Narrow spectrum penicillins (e.g. ampicillin)
- [ ] Anti-staphylococcal penicillins (e.g. oxacillin)
- [ ] Narrow-spectrum beta-lactamase/beta-lactamase inhibitor combinations (e.g. ampicillin-sulbactam)
- [ ] Broad-spectrum beta-lactamase/beta-lactamase inhibitor combinations (e.g. piperacillin-tazobactam)
- [ ] 1st generation cephalosporins
- [ ] 2nd generation cephalosporins
- [ ] 3rd generation cephalosporins
- [ ] 4th generation cephalosporins (e.g. cefepime)
- [ ] 5th generation cephalosporins (e.g. ceftaroline)
- [ ] Aztreonam
- [ ] Carbapenems
- [ ] Aminoglycosides
- [ ] Fluoroquinolones
- [ ] Macrolides
- [ ] Telithromycin
- [ ] Clindamycin
- [ ] Quinupristin/dalfopristin
- [ ] Tetracyclines
- [ ] Tigecycline
- [ ] Trimethoprim-sulfamethoxazole
- [ ] Polymixins (e.g., colistin)
- [ ] Daptomycin
- [ ] Glycopeptides (e.g. vancomycin)
- [ ] Oxazolidinones (e.g. linezolid)
- [ ] Fosfomycin
- [ ] Fidaxomicin
- [ ] Metronidazole
- [ ] Rifaximin
- [ ] Nitrofurantoin
- [ ]
- [ ] --------------------- Systemic Antifungals ----------------------
- [ ] Amphotericin B
- [ ] Echinocandins
- [ ] Fluconazole
- [ ] Ketaconazole
- [ ] Voriconazole
- [ ] Posaconazole
- [ ] Isovuconazole
- [ ] Flucytosine
- [ ] Griseofulvin
- [ ] Pentamidine
- [ ] Terbinafine
- [ ]
- [ ] --------------------- Antivirals ----------------------
- [ ] Acyclovir
- [ ] Cidofovir
- [ ] Famciclovir
- [ ] Fosamprenivir
- [ ] Foscarnet
- [ ] Ganciclovir
- [ ] Oseltamivir
- [ ] Peramivir
- [ ] Ribavirin
- [ ] Valacyclovir
- [ ] Valganciclovir
- [ ] Vidarabine
- [ ] Zanamavir
- [ ]
- [ ] --------------------- Other anti-infectives:----------------------
- [ ] Atovaquone
- [ ] Dapsone
- [ ] Ivermectin

☐ Nitazoxanide
☐ Paromomycin
☐ Praziquantel
☐ Pyrantel pamoate

Do you have a restricted formulary or require prior-authorization of antimicrobials? (e.g., certain drugs can only be used by specific services, not all drugs within a class are available)

○ Yes
○ No

Which of the following drugs do you require prior authorization?

- ☐ --------------------- All Antibiotics --------------------------
- ☐ Narrow spectrum penicillins (e.g. ampicillin)
- ☐ Anti-staphylococcal penicillins (e.g. oxacillin)
- ☐ Narrow-spectrum beta-lactamase/beta-lactamase inhibitor combinations (e.g. ampicillin-sulbactam)
- ☐ Broad-spectrum beta-lactamase/beta-lactamase inhibitor combinations (e.g. piperacillin-tazobactam)
- ☐ 1st generation cephalosporins
- ☐ 2nd generation cephalosporins
- ☐ 3rd generation cephalosporins
- ☐ 4th generation cephalosporins (e.g. cefepime)
- ☐ 5th generation cephalosporins (e.g. ceftaroline)
- ☐ Aztreonam
- ☐ Carbapenems
- ☐ Aminoglycosides
- ☐ Fluoroquinolones
- ☐ Macrolides
- ☐ Telithromycin
- ☐ Clindamycin
- ☐ Quinupristin/dalfopristin
- ☐ Tetracyclines
- ☐ Tigecycline
- ☐ Trimethoprim-sulfamethoxazole
- ☐ Polymixins (e.g., colistin)
- ☐ Daptomycin
- ☐ Glycopeptides (e.g. vancomycin)
- ☐ Oxazolidinones (e.g. linezolid)
- ☐ Fosfomycin
- ☐ Fidaxomicin
- ☐ Metronidazole
- ☐ Rifaximin
- ☐ Nitrofurantoin
- ☐
- ☐ --------------------- Systemic Antifungals ---------------------
- ☐ Amphotericin B
- ☐ Echinocandins
- ☐ Fluconazole
- ☐ Ketaconazole
- ☐ Voriconazole
- ☐ Posaconazole
- ☐ Isovuconazole
- ☐ Flucytosine
- ☐ Griseofulvin
- ☐ Pentamidine
- ☐ Terbinafine
- ☐
- ☐ --------------------- Antivirals ---------------------
- ☐ Acyclovir
- ☐ Cidofovir
- ☐ Famciclovir
- ☐ Fosamprenivir
- ☐ Foscarnet
- ☐ Ganciclovir
- ☐ Oseltamivir
- ☐ Peramivir
- ☐ Ribavirin
- ☐ Valacyclovir
- ☐ Valganciclovir
- ☐ Vidarabine
- ☐ Zanamavir
- ☐
- ☐ --------------------- Other anti-infectives:---------------------
- ☐ Atovaquone
- ☐ Dapsone
- ☐ Ivermectin

☐ Nitazoxanide
☐ Paromomycin
☐ Praziquantel
☐ Pyrantel pamoate

Do you have condition specific clinical practice guidelines or care process models implemented at your hospital?	◯ Yes<br>◯ No
List applicable conditions that have clinical practice guidelines/ care process models.	____________________
Does your hospital/ASP have surgical prophylaxis guidelines?	◯ Yes<br>◯ No
Does your hospital/ASP have an empiric antimicrobial selection guidance for common infections?	◯ Yes<br>◯ No
Does your hospital have an IV to oral switch program?	◯ Yes<br>◯ No
Are indications required when an antimicrobial is ordered?	◯ Yes<br>◯ No
Do the majority of your clinicians perform antimicrobial time-outs after 48-72 hours of a patient receiving an antimicrobial?	◯ Yes<br>◯ No
Does your ASP receive rapid diagnostic results to help make interventions?	◯ Yes<br>◯ No
Does your ASP utilize automatic stop-orders?	◯ Yes<br>◯ No
Does your ASP/Pharmacy perform therapeutic drug monitoring (TDM) for antimicrobials?	◯ Yes<br>◯ No
Which drugs do you perform therapeutic drug monitoring?	☐ Vancomycin<br>☐ Aminoglycosides<br>☐ Voriconazole<br>☐ Posaconazole<br>(Check all that apply)
Does your ASP perform dose adjustments/optimization?	◯ Yes<br>◯ No
Are pharmacists able to make ASP recommendations independent of a physician?	◯ Yes<br>◯ No
Does your ASP document their recommendations in the medical chart?	◯ Yes<br>◯ No
Is ASP documentation considered an official part of the medical record?	◯ Yes<br>◯ No

Computer Support

Please respond to the following questions as they apply to pediatric patients only.

Do you use an ASP module that is within your main electronic health record system? (eg. ASP module developed by Epic)?
○ Yes
○ No

Which EHR system is used? ______________________________

Does your hospital have a third party computer surveillance system (eg. Vigilanz, med-mined, Theradoc, Sentri7) that can help with ASP and/or Infection Control?
○ Yes
○ No

Which System?
☐ Vigilanz
☐ MedMined
☐ Sentri7
☐ Home-grown system
☐ Pathfinder
☐ Theradoc
☐ Other
(Check all that apply)

Do you use the system to help with antimicrobial stewardship?
○ Yes
○ No

If yes, describe how you use the system for antimicrobial stewardship.
____

Do you have condition specific order sets to influence antimicrobial prescribing?
○ Yes
○ No

Data Tracking and Reporting

Please respond to the following questions as they apply to pediatric patients only.

Does your hospital/ASP review antimicrobial use reports on a regular basis (e.g., monthly, quarterly, etc)?
○ Yes
○ No

If yes, do you monitor days of therapy per 1000 patient days?
○ Yes
○ No

How do you obtain your DOT per 1000 patient days? (click all that apply)
☐ PHIS
☐ eMAR
☐ computer surveillance system
☐ other
(Check all that apply)

Describe the other way you obtain your DOT.
____

Who do you share antimicrobial use data with?

- ☐ No one
- ☐ Hospital administration
- ☐ front-line clinicians
- ☐ P&T
- ☐ ASP committee
- ☐ other

(Check all that apply)

Describe the other way you share antimicrobial data.

____

Does your hospital/ASP review metrics related to antimicrobial cost? (e.g., cost per patient day, total antimicrobial pharmacy cost, etc)

- ◯ Yes
- ◯ No

Does your hospital develop an annual antibiogram?

- ◯ Yes
- ◯ No

Does your hospital/ASP monitor hospital acquired C. difficile rates?

- ◯ Yes
- ◯ No

Does your hospital collect the number or incidence of MDROs over a specific time frame (e.g., monthly, annually)?

- ◯ Yes
- ◯ No

Does your hospital monitor antimicrobial related adverse drug reactions?

- ◯ Yes
- ◯ No

Does your hospital/ASP monitor any other clinical outcomes?

- ◯ Yes
- ◯ No

Which other clinical outcomes does your hospital/ASP monitor?

____

Which of the following educational activities does your ASP perform? Check all that apply.

- ☐ Annual education to all prescribers (e.g. required online module)
- ☐ Lectures to trainees
- ☐ Grand Rounds
- ☐ Education targeting patients and families
- ☐ Other
- ☐ None of the above

(Check all that apply)

Describe the annual education you provide to all providers.

____

Describe the education you provide for patients and families.

____

Describe the other education you provide.

____
